# Prevalence of Malaria and Associated Factors among Under Five Febrile Children Attending Ener Amanuel Health Center, Southern Ethiopia: An Institution-based Cross-Sectional Study

**DOI:** 10.4314/ejhs.v35i5.2

**Published:** 2025-09

**Authors:** Sintayehu Tsegaye Tseha, Mamaru Ebabu, Temam Aberar

**Affiliations:** 1 Department of Biology, Arba Minch University, Arba Minch, Ethiopia; 2 Kose-Hidase Preparatory School, Enor-Ener and Meger Woreda, Ethiopia

**Keywords:** Associated factors, Ener Amanuel Health Center, Malaria prevalence, Under-five children

## Abstract

**Background:**

Malaria is a major public health problem in Ethiopia, including Enor-Ener Woreda. This study aimed to determine the prevalence and associated factors of malaria among febrile children under five years of age attending Ener Amanuel Health Center, Southern Ethiopia.

**Methods:**

A cross-sectional study was conducted at Ener Amanuel Health Center, located in Enor-Ener Woreda, from December 2022 to April 2023. A total of 218 febrile children under five years were enrolled. Blood samples were collected, and malaria was diagnosed using microscopy. Bivariate and multivariable logistic regression analyses were performed to identify factors associated with Plasmodium infection.

**Results:**

The prevalence of malaria was 21.1% in Enor-Ener Woreda, with P. falciparum being the predominant species (60.9%). Factors significantly associated with Plasmodium infection included proximity to mosquito breeding sites (P=0.025), not sleeping under insecticide-treated nets (ITNs) (P=0.021), and having beds unsuitable for ITN hanging (0.002).

**Conclusion:**

Malaria remains a significant public health concern in the study area. Key factors associated with infection include the presence of mosquito breeding sites and improper ITN use. Strengthening ITN utilization and eliminating mosquito breeding sites are essential to reduce the malaria burden among under-five children in the area.

## Introduction

Malaria remains a major public health problem in Sub-Saharan Africa, disproportionately affecting children under the age of five (1). According to the World Malaria Report, there were 249 million cases of malaria in 2022 compared to 244 million in 2021, with Africa accounting for 94% of all cases and 95% of malaria-related deaths. Children under five years of age represented about 78% of all malaria deaths in Africa (1). Moreover, malaria is a major public health challenge that hinders national development and productivity (2).

In Ethiopia, about 52 million people (68% of the population) live in malaria risk zones, mostly below 2,000 meters of elevation (3). Children under five are the age group most disproportionately affected, largely due to their immature immune systems (4). Malaria in this group can lead to severe complications such as brain damage, respiratory distress, mental impairment, and high fever (5). According to the Enor-Ener Woreda Health Office report, malaria is one of the leading public health concerns in Enor-Ener Woreda. However, no studies have been conducted at Ener Amanuel Health Center in Enor-Ener Woreda to assess the prevalence of malaria and associated factors in children under five. Therefore, this study aimed to determine the prevalence of malaria and associated factors among children under five at Ener Amanuel Health Center, Southern Ethiopia.

## Materials and Methods

**Study area**: The study was conducted at Ener Amanuel Health Center, located in Enor-Ener Woreda, about 211 km southwest of Addis Ababa, the capital of Ethiopia. Enor-Ener Woreda is bordered by Enamor Woreda in the north, Hadiyia in the south, Yem Ley Woreda in the west, and Endegagn Woreda in the east. It is part of the Gurage Zone in the Central Ethiopia Regional State and comprises 24 rural and 3 urban kebeles. The Woreda lies between 900 and 3,200 meters above sea level, with mean annual rainfall ranging from 801 mm to 1,400 mm and monthly temperatures between 12.6°C and 25°C. The main rainy season extends from June to September, while a minor rainy season occurs from March to April. Enor-Ener Woreda has three health centers, one private clinic, and several pharmacies. According to the Enor-Ener Woreda Health Office annual report, malaria is one of the leading diseases in the area.

**Study design**: An institution-based cross-sectional study was conducted from December 2022 to April 2023 to assess malaria prevalence and associated factors among 218 febrile children under five attending Ener Amanuel Health Center, Enor-Ener Woreda. In addition, retrospective data were collected to examine malaria prevalence trends among children under five over the past five years (2018–2022).

**Study variables**: The dependent variable in this study is malaria prevalence among febrile children under five. The independent variables are: availability of insecticide-treated nets (ITNs), sleeping under ITNs, use of indoor residual spraying (IRS), age, gender, place of residence (urban/rural), educational status, knowledge of malaria, availability of drainage systems, mosquito breeding sites near residences, distance from the health center, housing structure, and socio-economic status.

**Source and study population**: The source population consisted of all patients visiting Ener Amanuel Health Center during the study period. The study population comprised febrile children under five years of age (body temperature ≥ 38 °C) attending the health center.

**Inclusion criteria**: Febrile children under five years with body temperature > 38 °C, whose parents or caregivers provided informed consent.

**Exclusion criteria**: Critically ill children and those who had taken anti-malarial drugs prior to the study were excluded.

**Sample size determination**: The sample size (n) was determined using a single population proportion formula. A previous study in Wolkite, Southern Ethiopia, reported a malaria prevalence (P) of 15.2% among children under five (6). With a 95% confidence level, a margin of error (d) of 0.05, and a standard score (Zα/2) of 1.96, the calculated sample size was 198. To account for potential non-response, 10% (20 participants) was added, giving a final sample size of 218.

**Data collection procedures and tools**: Febrile children were assessed by clinicians and referred for laboratory diagnosis. After obtaining informed consent, caregivers were interviewed using a standardized, pre-tested, interviewer-administered questionnaire in English, translated into Amharic and Guragigna. The questionnaire covered socio-demographic, economic, and environmental factors. Data collectors, who were trained health professionals fluent in local languages, received one day of training covering study objectives, interviewing techniques, and respondent interaction. Daily supervision was provided by the principal investigator.

Blood samples were collected via finger prick following the Ministry of Health's National Malaria Guidelines (7). Thick and thin blood smears were prepared. After drying, thin films were fixed with methanol, and all slides were stained with 10% Giemsa. Microscopic examination was performed by trained laboratory technicians at 100x magnification. A total of 100 microscopic fields were examined per slide, following WHO protocol (8). Plasmodium species identification was done on thin films, while thick films were used to confirm presence or absence of parasites. A slide was considered positive if one or more Plasmodium asexual stages were detected (9).

**Quality assurance**: All laboratory equipment and materials (slides, cover slips, microscopes, Giemsa stain) were checked before use. Each slide was cross-checked by a skilled professional at Ener Amanuel Health Center to minimize misidentification and discrepancies.

**Data analysis**: Data were analyzed using SPSS version 25. Bivariate and multivariable logistic regression analyses were conducted to identify factors associated with malaria infection. Variables with a p-value < 0.25 in bivariate analysis were included in the multivariable model. Adjusted odds ratios (AORs) with 95% confidence intervals (CIs) were calculated, and variables with p-values < 0.05 were considered statistically significant.

**Ethical considerations**: Ethical clearance was obtained from the Institutional Ethics Review Board of Arba Minch University. After explaining the study objectives, procedures, risks, and benefits, written informed consent was obtained from parents or caregivers before data collection. In line with government recommendations, all children who tested positive for malaria received free antimalarial treatment.

## Results

**Socio-demographic characteristics of children under five years:** A total of 218 febrile children under five years of age were included in the study. The majority were male, accounting for 156 (71.6%). The mean age of the children was 31.2 ± 0.804 months, ranging from 12 to 59 months. Most children (61%) were in the age group of 37–59 months. In addition, the majority (72.0%) resided in rural areas (Table 1).

**Table 1:** Socio-demographic characteristics of under five children in Ener Amanuel Health Center, Enor-Ener Woreda, Southern Ethiopia, December 2022 to April 2023

Characteristics	Number (%)
Sex	
Male	156 (71.6)
Female	62 (28.4)
Age in Month	
12-24	41(18.8)
25-36	44 (20.2)
37-59	133 (61.0)
Residence	
Urban	61 (28.0)
Rural	157 (72.0)

**Prevalence of malaria species in children under five years**: The overall prevalence of malaria in the study area was 21.1% (95% CI = 20.3–21.9), with *P. falciparum* being the predominant species (60.9%). The lowest prevalence was observed among the youngest children (12–24 months) (P = 0.009). Malaria prevalence was significantly higher among children living in rural areas 41 (89.1%) compared to those in urban areas (P = 0.004) (Table 2).

**Table 2:** *Plasmodium* infection among febrile under-five children in Ener Amanuel Health Center, Enor-Ener Woreda, Southern Ethiopia, December 2022 to April 2023

Variables		N (%)	Positive n (%)	*P.falciparum* n (%)	*P. vivax* n (%)	Mixed n (%)	Negative n (%)	P-value
Sex	Male	156(71.6)	26(56.5)	14(50)	9(60)	3(100)	130(75.6)	0.011
	Female	62(28.4)	20(43.5)	14(50)	6(40)		42(24.4)	
Age, (in months)	12-24	41(18.8)	4(8.7)	3(10.7)	1(6.6)	–	37(21.5)	0.009
	25-36	44(20.2)	16(34.8)	10(35.7)	3(20)	3(100)	28(16.3)	
	37-59	133(61.0)	26(56.5)	15(53.6)	11(73.3)	–	107(62.2)	
Residence	Urban	61 (28.0)	5(10.9)	3(10.7)	2(13.3)	–	56(32.6)	0.004
	Rural	157(72.0)	41(89.1)	25(89.3)	13(86.7)	3(100)	116(67.4)	
Total		218(100)	46(21.1)	28(60.9)	15(32.6)	3(6.5)	172(78.8)	

**Determinant factors for malaria positivity**: Multivariable logistic regression analysis revealed that not sleeping under ITNs (AOR = 2.877, 95% CI = 1.173-7.056, P=0.021), the presence of mosquito breeding sites near residences (AOR = 2.785, 95% CI = 1.135-6.736, P=0.025), and the number of beds unsuitable for ITN hanging (AOR = 2.054, 95% CI = 1.297-3.253, P=0.002) were significantly associated with *Plasmodium* infection in the study area (Table 3).

**Table 3:** Bivariate and multivariable analysis of factors associated with malaria positivity among febrile under five children, in Ener Amanuel Health Center, Enor-Ener Woreda, Southern Ethiopia, December 2022 to April 2023

Variables		Total N (%)	Positive n (%)	Negative n (%)	COR (95%CI)	AOR (95%CI)
Is your house near the health center	Yes	71(32.6)	21(45.7)	50(29.1)	2.050(1.052-3.993)	1.788(0.855-3.738)
	No	147(67.4)	25(54.3)	122(70.9)	1	1
Do you have ITNs	Yes	72(33.0)	22 (47.8)	50(29.1)	2.237(1.150-4.352)	2.052(0.982-4.290)
	No	146(67.0)	24 (52.2)	122(70.9%\)	1	1
Under-5 children sleep under ITNs	Yes	36(16.5)	12(26.1)	24(14.0)	2.176(0.991-4.781)	2.877(1.173-7.056)*
	No	182(83.5)	34(73.9)	148(86.0)	1	1
No. of beds suitable for ITN hanging	>2	5(2.3)	3 (6.5)	2(1.2)	2.103(1.370-3.229)	2.054(1.297-3.253)*
	2	14(6.4)	7 (15.2)	7(4.1)		
	1	73(33.5)	17(37.0)	56(32.6)		
	0	126(57.8)	19(41.3)	107(62.2)	1	1
Is there a mosquito breeding site near residence	Yes	152(69.7)	38 (82.6)	114(66.3)	2.417(1.059-5.516)	2.785(1.135-6.836)*
	No	66(30.3)	8(17.4)	58(33.7)	1	1
Has IRS applied to the home in the last five months	Yes	53(24.3)	5(10.9)	48(27.9)	0.315(0.117-0.845)	0.372(0.133-1.043)
	No	165(75.7)	41(89.1)	124(72.1)	1	1

AOR: adjusted odds ratio, CI=Confidence interval, COR: Crude odds ratio, ITN: Insecticide treated nets, IRS: Indoor Residual spray.

* P<0.05 statistically significant

## Discussion

This study demonstrated that malaria remains a public health problem among children under five in Enor-Ener Woreda. The overall prevalence of malaria among children visiting Ener Amanuel Health Center was 21.1% (95% CI = 20.3–21.9). The study was conducted from December 2022 to April 2023, which may not reflect the true annual prevalence, as malaria transmission is typically higher during the main transmission season (September to December).

The prevalence observed in this study is comparable to findings from other parts of Ethiopia, including Arsi Negele (22.8%) (10), Arba Minch Zuria District (22.1%) (11), and East Shewa Zone of Oromia (20.5%) (12). However, the results differ from reports in other areas, such as East Shewa Zone (25%) (13), Benna Tsemay District (6.1%) (14), Armachiho District (18.4%) (15), Wogera District (8.7%) (16), and Benishangul-Gumuz Region (3.9%) (17). These differences may be attributed to variations in the strength and effectiveness of malaria prevention and control measures across the different study areas.

In this study, *P. falciparum* and *P. vivax* were the only species detected, with *P. falciparum* accounting for 60.9% of infections. This finding aligns with studies conducted in Dembia District (18), Kombolcha (19), Dembecha (20), and Ataye (21). However, it contrasts with research from Dila, Southern Ethiopia, where *P. vivax* was reported as the predominant species (22).

Children living near mosquito breeding sites were more likely to test positive for malaria compared to those living farther away. This finding is consistent with studies conducted in Dembia (23), Gedeo Zone (24), and HadiyaZone (25).

The study also showed that children who slept under insecticide-treated nets (ITNs) had a lower risk of malaria infection compared to those who did not. This is in agreement with earlier studies from Gedeo Zone, Southern Ethiopia (24), and the Benishangul-Gumuz Region, Southwest Ethiopia (17).

This study has some limitations. First, data were collected only from health facilities, which may underestimate the true burden of malaria in under-five children in the community. Second, although several risk factors for malaria transmission were assessed, the effects of factors such as maternal education and household conditions were not examined. Therefore, further community-based studies are recommended to establish the actual prevalence and determinants of malaria in Enor-Ener Woreda.

In conclusion, malaria prevalence in Enor-Ener Woreda was 21.1% in 2023, with *P. falciparum* being the dominant species. Significant factors associated with malaria infection included proximity to mosquito breeding sites, not sleeping under ITNs, and lack of beds suitable for ITN hanging. Strengthening ITN utilization and eliminating mosquito breeding sites are therefore crucial to reducing the malaria burden among children under five in this area. Furthermore, community-based studies are needed to better determine the prevalence and risk factors for malaria among under-five children in Enor-Ener Woreda.
